# Entrectinib-Induced Sinus Bradycardia in a Patient With Metastatic Non–Small Cell Lung Cancer

**DOI:** 10.1016/j.jaccas.2026.107369

**Published:** 2026-03-16

**Authors:** Azita H. Talasaz, Anne J. Goldberg, Catherine A. Shu, Gabriel T. Sayer, Nir Uriel, Jayant K. Raikhelkar

**Affiliations:** a Department of Pharmacy, New York Presbyterian Hospital/Columbia University Medical Center, New York, New York, USA; b Department of Medicine, Division of Cardiology, Columbia University Vagelos College of Physicians and Surgeons, New York, New York, USA; c Division of Hematology/Oncology, Columbia University Medical Center, New York, New York, USA

**Keywords:** cardio-oncology, entrectinib, lung cancer

## Abstract

**Background:**

Targeted therapies like tyrosine kinase inhibitors have significantly improved outcomes in non–small cell lung cancer. Entrectinib, a multikinase inhibitor, showed efficacy in *ROS1* fusion-positive NSCLC, including intracranial metastases. Although generally well tolerated, rare but serious cardiac adverse effects have been reported.

**Case Summary:**

We report a 51-year-old woman with *ROS1*-mutant metastatic non–small cell lung cancer who developed symptomatic sinus bradycardia during entrectinib therapy. After disease progression on crizotinib, lorlatinib, and reprotrectinib, she was started on entrectinib. Within 1 week, she developed marked bradycardia (38-48 beats/min) with lightheadedness. Electrocardiogram and echocardiography confirmed sinus bradycardia with mildly reduced ejection fraction. Entrectinib was discontinued, and symptoms resolved with heart rate normalization.

**Take-Home Messages:**

Sinus bradycardia may be a rare but significant side effect of entrectinib, possibly related to ALK inhibition. Cardiac monitoring and multidisciplinary management are essential when initiating therapy with *ALK/ROS1* inhibitors.

## Visual Summary

**Figure:**
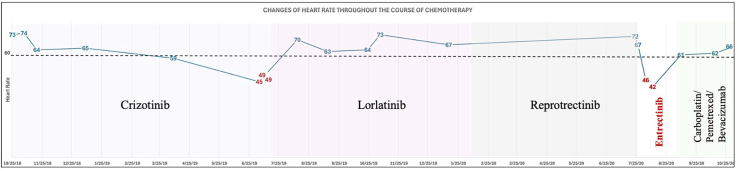

**Visual Summary:**
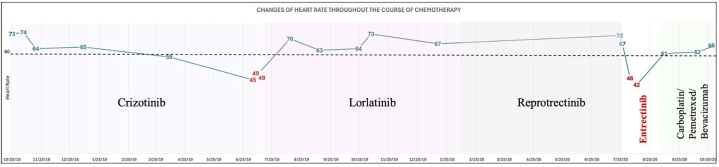
Changes of Heart Rate Throughout the Course of Chemotherapy The timeline for each chemotherapy regimen is shown alongside corresponding heart rate measurements. The dotted line indicates a heart rate <60 beats/min.

Targeted therapies, such as tyrosine kinase inhibitors, have transformed cancer treatment, improving survival. Entrectinib, a recently approved multikinase inhibitor, targets ROS1 gene fusions (in addition to tropomyosin receptor kinase [*TRK*] *A*, *B*, and *C*, and *ALK*).^1^ *ROS1* fusions are present in 1% to 2% of non–small cell lung cancer (NSCLC) and have been associated with increased risk of intracranial metastasis.^2^^,^^3^ Although crizotinib is approved for the treatment of patients with advanced *ROS1*-fusion positive NSCLC, its suboptimal central nervous system penetration makes it a poor choice for these patients.^4^ Clinical trials (ALKA-372-001, STARTRK [Studies of Tumor Alterations Responsive to Targeting Receptor Kinases]-1, STARTRK-2, and STARTRK-NG [Studies of Tumor Alterations Responsive to Targeting Receptor Kinases ‐ Next Generation]) using entrectinib in patients with locally advanced or metastatic *ROS1* fusion-positive NSCLC have shown significant clinical benefit, including efficacy in patients with central nervous system metastases.^1^^,^^5^ Although generally well tolerated, the most frequent serious treatment-related adverse events were nervous system and cardiac disorders. Three cases of cardiac tamponade, cardiogenic shock, and myocarditis have been reported in clinical trials.^6^ To our knowledge, this is the first report of sinus bradycardia associated with the use of entrectinib in a patient with a *ROS1* fusion-positive NSCLC.

**Take-Home Messages**

• Sinus bradycardia represents a rare but clinically significant adverse effect of entrectinib, potentially attributable to ALK-mediated modulation of cardiac conduction pathways.
• Previous bradycardia with other *ALK* inhibitors may be a risk factor.
• Rigorous cardiac surveillance and interdisciplinary coordination between oncology and cardiology are imperative to optimize therapeutic outcomes and mitigate cardiovascular risks during *ALK/ROS1* inhibitor treatment.

## History of Presentation

A 51-year-old woman with stage IV *ROS1*-mutant lung adenocarcinoma, metastatic to the brain, pleura, pericardium, and liver was started on crizotinib as first-line therapy. With evidence of progression after 8 months of treatment and profound bradycardia (heart rate [HR]: 41 beats/min), lorlatinib was initiated with partial improvement in cerebellar lesions. Due to further progression, she underwent gamma knife therapy for larger lesions and was referred for a reprotrectinib trial;^7^ however, her brain disease worsened after being in this study for 3 months. She eventually was treated with whole brain radiation and started on entrectinib 400 mg daily. Sinus bradycardia was noted in the first week of treatment and the patient was referred to cardio-oncology clinic.

## Investigations

**Figure 1:**
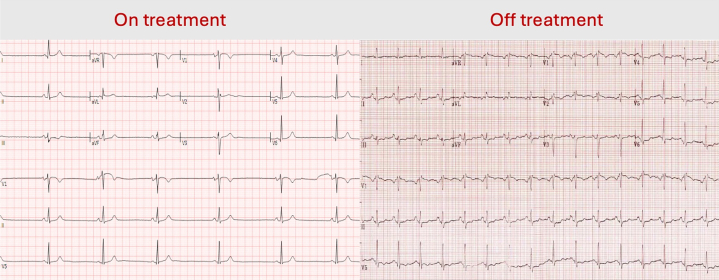
Electrocardiogram of Patient on and Off Treatment With Entrectinib The left panel shows the patient's electrocardiogram while she was on treatment with entrectinib, and the right panel shows the patient's electrocardiogram while treatment of entrectinib was discontinued.

Serial electrocardiograms revealed marked sinus bradycardia (38-48 beats/min) low voltage, with incomplete right bundle branch block and left atrial abnormality, associated with reported lightheadedness and dizziness (Figure 1). Physical examination was unremarkable. Echocardiography showed mild left ventricular dysfunction with the left ventricular ejection fraction of 50% to 55% without valvular dysfunction or regional wall motion abnormalities. High-sensitivity troponin I (22 ng/L) and N-terminal pro–B-type natriuretic peptide (218 pg/mL) were mildly elevated. All other laboratory results were within normal limits.

## Management

Entrectinib was discontinued only after 1 month of treatment due to severe neuropsychiatric symptoms and symptomatic bradycardia. An event monitor was placed to assess HR for 1-week post-treatment. HR ranged from 50 to 120 beats/min, with an average of 79 beats/min, and premature atrial contractions accounted for <1% of the total.

## Outcome and Follow-Up

**Figure 2:**
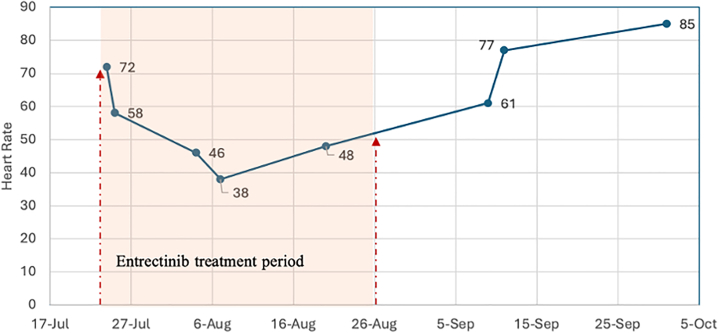
Changes in Heart Rate Over Time of Treatment (On and Off Entrectinib) The highlighted section shows the changes in heart rate while the patient was on treatment with entrectinib. The dots show the heart rate on each visit. The patient's heart rate improved after the discontinuation of entrectinib treatment.

During follow-up visits, the patient's HR improved to 77 and 85 beats/min, respectively (Figure 2). She reported no further episodes of lightheadedness or dizziness, indicating resolution of her previous symptoms. The patient continued treatment with carboplatin, pemetrexed, and bevacizumab followed by maintenance therapy with pemetrexed and bevacizumab. She developed hypertension secondary to bevacizumab, which was managed with lisinopril 40 mg daily. She eventually underwent a right thoracotomy lobectomy and bronchial plasty with partial decortication and is otherwise doing well after 5 years of treatment.

## Discussion

Entrectinib is a multikinase inhibitor targeting *ROS1*, *ALK*, and pan-*TRK*, primarily used in the treatment of NSCLC. It is generally well tolerated, with common adverse effects including dysgeusia, vertigo, constipation, and fatigue, typically of grades 1 to 2 mostly related to *TRK* inhibition. However, there have been rare reports of cardiac toxicities, including heart failure which occurred in 3.4% of patients in phase I/II trials with 0.3% grade 4 myocarditis.^8^^,^^9^ Fulminant myocarditis associated with cardiogenic shock has also been reported recently.^10^ These events occur mostly early in treatment within the first month. This report describes a case of sinus bradycardia, potentially related to *ALK* inhibition, which resulted in the discontinuation of entrectinib treatment after 1 month of treatment. A systematic review of *ALK* inhibitor–associated bradycardia in randomized controlled trials suggests that this adverse effect, although variable in incidence, may be a class effect and was accompanied with dizziness.^11^ This bradycardia may stem from off-target chronotropic and dromotropic effects on the cardiac conduction system, and possible autonomic modulation.^12^ QTc prolongation is another reported conduction abnormality with entrectinib that warrants monitoring due to its arrhythmic potential.^13^ In patients with severe or symptomatic bradycardia associated with the use of *ALK* inhibitors, it is recommended to hold treatment until the HR exceeds 60 beats/min. On HR recovery, treatment with the *ALK* inhibitor can be restarted at a reduced dose with the option for permanent discontinuation in case of life-threatening event or if no other contributing causes have been identified.^11^ In our patient's case, entrectinib was discontinued in consultation with the oncology team due to concurrent neuropsychiatric symptoms. Notably, the patient had also experienced bradycardia while on crizotinib, indicating a propensity to bradycardia with *ALK* inhibition. She subsequently resumed systemic chemotherapy.

## Conclusions

Profound sinus bradycardia is a possible adverse effect of entrectinib. Providers should be vigilant of this in addition to previously described cardiotoxicities which include QT prolongation and heart failure. A multidisciplinary approach is crucial for optimizing cancer treatment while ensuring cardiovascular safety.

## Funding Support and Author Disclosures

Dr Shu has received personal fees from AstraZeneca, Genentech, EMD Serono, and JNJ. All other authors have reported that they have no relationships relevant to the contents of this paper to disclose.
